# Development of the abdominal musculature in the chicken embryo

**DOI:** 10.1111/joa.70113

**Published:** 2026-01-22

**Authors:** Margarethe Draga, Luke Orth, Julia Khabyuk, Vanessa Holtwick, Johanna Heinen, Felicitas Pröls, Martin Scaal

**Affiliations:** ^1^ Institute of Anatomy and Developmental Biology, Faculty of Medicine and University Hospital Cologne University of Cologne Cologne Germany

**Keywords:** abdominal, chicken, development, embryo, muscle, muscle fiber, myotome, somite

## Abstract

The abdominal musculature is of prime importance for stabilization and flexion movements of the trunk, and of high clinical relevance in hernia surgery. Despite a number of severe congenital malformations involving defective abdominal muscle formation, little is known about abdominal muscle development during embryogenesis. Here, we used the chicken model system to investigate anatomy, morphogenesis, segmental origin, and fiber formation of the abdominal musculature. The specific anatomy of the chicken abdominal musculature was first determined by macroscopic dissections of adult specimens. We then describe the sculpting of the external oblique, internal oblique, transversus, and rectus muscles from a uniform hypaxial muscle blastema on the basis of whole‐mount embryos and sections. We show that abdominal muscles arise from somites 24–28, that all abdominal muscles receive muscle cells from multiple somites, and that somites 25 and 26 provide the major source of abdominal muscle cells. We find that the contribution of individual somites to distinct muscle portions is heterogeneous, with a roughly segmental arrangement of muscle fibers in the transversus muscle, and a random mix of fibers of different segmental origin in the rectus muscle. We furthermore show that despite extensive fiber mixing, there is no fusion between fibers of different segments, so that the segmental identity of individual fibers in the abdominal muscle sheets is maintained. We present abdominal muscle formation as a paradigm for the development of large, segment‐crossing trunk muscles from segmental myotomal anlagen.

## Graphical abstract

In this study, we investigate anatomy, morphogenesis, segmental origin, and fiber formation of the abdominal musculature in the chicken embryo. We show that abdominal muscles arise from somites 24 to 28, and that the segmental identity of individual fibers in the abdominal muscle sheets is maintained.

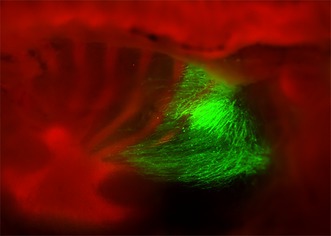

## 1 INTRODUCTION

The abdominal wall musculature is well known in human anatomy to enable ventral flexion and mediolateral rotation of the trunk, as well as to provide stabilization of the vertebral column and abdominal pressure to support respiration. It consists of the three‐layered lateral abdominal muscles, *M. obliquus externus abdominis* (external oblique muscle), *M. obliquus internus abdominis* (internal oblique muscle), and *M. transversus abdominis* (transversus muscle), which are bilaterally interconnected by the fibrous rectus sheath and thus act as a functional entity. Moreover, within the rectus sheath, the paired *M. rectus abdominis* (rectus muscle) extends in longitudinal direction between the thorax and the pelvis (Standring, 2021). Together, abdominal muscles and aponeuroses form a firm yet flexible envelope which covers the abdominal viscera on the ventral and lateral aspect in the absence of a skeletal scaffold. From a clinical point of view, understanding the anatomy of the abdominal wall is crucial for hernia repair and abdominal wall reconstruction, being among the most frequent surgical interventions altogether (Kim et al., 2025; Pogson‐Morowitz et al., 2024; Rosenberg et al., 2025).

Despite the functional and clinical importance of the abdominal wall, surprisingly little is known about its development. Developmental defects in abdominal wall formation lead to increasingly diagnosed, severe malformations including gastroschisis and omphalocele (Khan et al., 2022; Marquart et al., 2024; Pontes et al., 2025), and specific defects of the abdominal musculature are seen in the Prune Belly Syndrome (Conegundes et al., 2025; Hassett et al., 2012). To fill the gap of knowledge on abdominal wall development, more basic research in animal model systems is required. In mammalian model systems like the rat, the architecture of the abdominal wall is similar to the human situation (Brown et al., 2010), but embryos are difficult to access for in vivo experimental manipulation. Here, we use the chicken embryo as a model system to explore the morphogenesis of the abdominal musculature, as the embryos are easily accessible for cell lineage experiments and well suited for further functional studies on the molecular regulation of abdominal wall development.

In birds, the nomenclature of the abdominal muscles is the same as in mammals, even though the muscle topography is slightly different in a number of aspects due to the adaptations of thorax and pelvis to flight and the specific mode of avian respiration. Avian abdominal muscles are relatively thin muscle sheets, forming a delicate muscular and aponeurotic covering of the abdominal cavity between the prominent sternum and the pelvis, which is partly hidden by the massive pectoral and femoral muscle groups (Gadow, 1891; Starck, 1979). Literature on avian abdominal muscle anatomy is scarce. In the older literature, the work by Nishi (1938) on vertebral trunk muscles addresses avian abdominal muscles only very briefly; the same holds true for the reference for avian myology by George & Berger (1966). Gadow and Selenka (Gadow, 1891) provide detailed descriptions of avian abdominal muscles in general, with some specific traits in selected species, but without illustrations. Shufeldt (1890) provides a detailed account on the musculature of the raven. More recent references for avian anatomy like Schummer (1973), König and Liebich (2001) and Baumel (1993) contain descriptions of origin, insertion, and innervation of avian muscles with special reference to the chicken, yet with partially conflicting information but lack illustrations. The illustrated atlas by Ghetie (1976) on the anatomy of chicken and other domestic birds presents detailed drawings of avian musculature in a number of topographic regions, but with insufficient depictions of abdominal muscles. To be able to clearly identify the emerging abdominal muscles during chicken embryogenesis, we performed dissections of adult chicken specimens which we combined with a review of the consensus data on chicken muscle anatomy from the literature and visualized the individual abdominal muscle morphology in schematic drawings corresponding to our embryo preparations.

The embryonic origin of the abdominal musculature has been controversial in the older literature, with data supporting somatopleural origin (His, 1868; Straus Jr. & Rawles, 1953) versus somitic origin (Bardeen, 1900; Engert, 1900; Fischel, 1885) of these muscles. More recent experimental data in the chicken embryo using variable cell tracking techniques have definitively shown that the abdominal muscles, like the intercostal muscles, solely derive from the somites, whereas the muscle‐associated connective tissue of the ventrolateral body wall is of somatopleural origin (Chevallier, 1975; Christ et al., 1978, 1981, 1983; Pinot, 1969; Seno, 1961). Beyond this, Engert (1900) and Christ et al. (1983) have provided detailed descriptions of abdominal muscle development in the chicken embryo based on the analysis of transverse sections of embryos after histological staining with borax carmine, and quail‐chick chimerization of somitic subcompartments, respectively. They conclusively found that the abdominal muscles are formed by cells from the primary hypaxial myotomes as well as from cells of the deepithelializing ventrolateral dermomyotomal lips. These cells form muscle tissue, which is still segmental at HH‐stage 24 but becomes confluent between segments from HH‐stage 25 onwards, thus forming a non‐segmented muscle mass. Within this initially uniform abdominal muscle mass, invasion of somatopleural mesenchyme creates septa which divide the muscular tissue into distinct muscular compartments that anticipate the prospective anatomical abdominal muscles. However, both studies were limited to the analysis of sections and do not provide sufficient information on three‐dimensional muscle morphogenesis. Probably owing to this, both studies differ in their results regarding the timing of muscle formation. Here, we studied the morphogenesis of the abdominal muscles by using whole‐mount embryos of consecutive stages in combination with transverse sections, both stained with muscle markers, to get an integral concept of abdominal muscle morphogenesis.

The segmental origin of the abdominal muscles in the chicken embryo is also controversial in the literature, depending on the technique used to label the somites of origin which give rise to the myogenic cells. Seno (1961) used carbon particles to label somites and their derivatives, and identified somites 26 and 27 to be the only origin of abdominal muscles. Chevallier (1979) used the quail‐chick chimerization technique and found the abdominal muscles originating from somites 27 to 29. Rees et al. (2003) used replication‐deficient lacZ‐encoding retroviruses injected into somites to identify the hind limb muscle lineage and found labelled cells from somites 26 to 29 in abdominal muscles. To clarify these conflicting results, we here addressed the somitic origin of the abdominal muscles by electroporating muscle‐specific in vivo markers into individual somites to visualize their contribution to abdominal muscles in high resolution.

All trunk muscles originate from segmental myotomes which are the muscular compartments of the somites. This original muscular segmentation is obviously preserved in the intercostal muscles, which form stripes of muscle with longitudinally arranged muscle fibers between the ribs that still resemble the embryonic myotomes in the adult (Khabyuk et al., 2022). Contrary to this, the long back muscles and the abdominal muscles lose their overt segmental morphology during muscle morphogenesis and stretch over a considerable distance along the vertebral column and between pelvis and sternum, respectively. In both cases, it is unknown whether the segmental origin of multisegmental muscles is maintained by the respective muscle fibers, or if the muscle fibers of these muscles mix at random. We addressed this here for the abdominal muscles by labelling neighboring somite cells with muscle‐specific expression constructs of different color, and monitoring their respective arrangement in the abdominal muscle sheets.

In large muscles, individual muscle fibers can attain considerable length, by far exceeding the extent of the vertebral motion segments, which represent the original somite‐derived segmentation of the body wall. The myotomal muscle fibers of the trunk muscles are of dual origin. The first muscle fibers forming the primary myotome involute from the dermomyotomal lips of the early somites (Gros et al., 2004). These primary myotomal cells are fiber‐like, mononucleated muscle cells, which span the craniocaudal extent of the respective segment. In a second step, Pax7‐positive cells emerge from the central dermomyotome after epithelial‐to‐mesenchymal transition and “parachute” in between the scaffold of primary myotomal cells, where they form a second muscle cell pool called resident muscle precursor cells (Gros et al., 2005). Subsequently, the primitive muscle anlagen grow extensively. In the embryo, this process can, in principle, be achieved by the growth of mononucleate primary myotomal muscle cells to multinucleate fibers via fusion, or by fusion of resident, dermomyotome‐derived muscle precursor cells. Fusion of primary myotomal cells with resident muscle precursors is rare, while fusion of primary myotomal cells with other primary myotomal cells, and likewise fusion of resident muscle precursors with each other, are the major mode of muscle fiber growth in early trunk muscles (Sieiro‐Mosti et al., 2014). These processes have so far only been studied in individual myotomes, that is within a single segment, and in limb muscles (Sieiro‐Mosti et al., 2014). How segment‐crossing, long muscle fibers in the trunk develop are still unclear. In order to decide whether muscle fibers of the abdominal muscles only arise from fusion of cells from the same segment, or also from fusion with cells from neighboring segments, we again labelled neighboring somite cells with muscle‐specific expression constructs of different color and checked for the existence of muscle fibers of mixed segmental origin under the confocal microscope.

In summary, in this work, we describe the anatomy and morphogenesis of chicken abdominal muscles both in whole‐mount embryos and in sections, and studied the segmental origin and composition of the abdominal muscles. We found that the abdominal muscles arise from the hypaxial myotomes by extensive muscle fiber reorientation and growth, and that abdominal muscle precursors emerge from somites 24–28 to different extents, with somites 25 and 26 being the major source of abdominal muscles. We show that the segmental origin of muscle fibers is preserved in the transversus muscle along its craniocaudal extent but is lost in other abdominal muscles. We furthermore show that in abdominal muscles of mixed segmental origin, the individual muscle fibers develop from precursors of a single segment, and that muscle precursors of neighboring segments do not mix during muscle cell fusion.

## 2 METHODS

### 2.1 Adult chicken dissection

Three adult chicken cadavers (1 male, 2 female) which had not been disemboweled after slaughtering were purchased from local breeders and dissected using anatomical scalpels and forceps to demonstrate the abdominal musculature. Photographs were taken with a digital camera and optimized with Photoshop, Adobe Inc., to replace the background with black color for the sake of clarity.

### 2.2 Embryos

For all experiments, fertilized chicken eggs (Gallus gallus domesticus, White Leghorn, Lohmann LSL weiß) provided by academic and commercial breeders (Lehr‐ und Forschungsstation Gut Frankenforst, Agricultural Faculty, University of Bonn; LSL, Lohmann Deutschland GmbH & Co KG, Ankum) were incubated at 37.5°C and at 55% humidity until they reached the required age according to Hamburger and Hamilton stages (HH‐stages) (Hamburger & Hamilton, 1951). Embryos were isolated, decapitated, and cut in half in the para‐sagittal plane. To enable a medial view of the abdominal muscles, all internal organs were carefully removed with the use of forceps.

### 2.3 Somite counting

In the embryonic stages manipulated here, the first visible somite represents the somite of segment 2, as the first somite is cranially continuous with the unsegmented cranial paraxial mesoderm without cranial segment border and disappears morphologically around the 7–10 somite stage (Couly et al., 1993; Williams, 1910). Thus, our somite countings count the most cranially visible complete segment, that is, segment cranially and caudally delimited by an intersomitic cleft, as somite 2.

**FIGURE 1:**
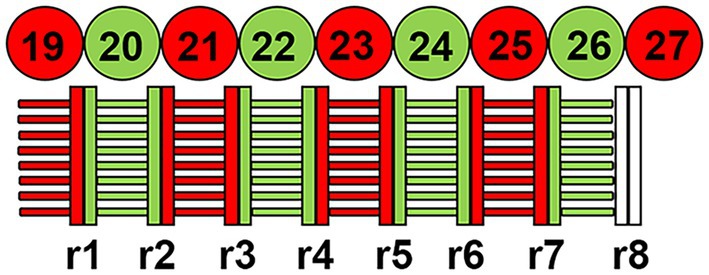
Verification of somite counting based on segmental identity according to Burke et al. (1995). Numbered circles represent somites with segment number in alternating color code, vertical bars represent ribs r1–r7 (variable eighth rib: r8) arising from the respective somites, and horizontal bars represent intercostal muscles arising from the respective somites. According to the resegmentation concept, an individual rib shaft is formed by cells of two neighboring somites, whereas intercostal muscles remain segmental. This enables precise assignment of an electroporated somite to the correct segment number.

It is acknowledged that counting somites in embryos of advanced stages is technically very difficult due to embryo rotation and increasing density of subectodermal tissue (Burke et al., 1995). As precise counting was crucial in this study, we resorted to the somite regionalization concept established by Burke et al. (1995) which is based on Hox gene expression and identifies somite 19 as the first and somite 26 as the last thoracic segment in chicken. Consequently, as somite 26 represents the thoraco‐lumbal boundary, this implies that somite 25 is the caudalmost somite giving rise to intercostal muscle, and somite 26 is the caudalmost somite giving rise to rib cartilage (Figure 1). We thus confirmed our counting method by co‐staining selected electroporated embryos with antibodies against muscular desmin and Sox9 expressed in rib cartilage (see Section 3).

### 2.4 Immunohistochemistry

Embryos of HH‐stages 24–32 were isolated, prepared as described above, fixed in 4% paraformaldehyde at 4°C overnight, washed in 1× PBS, permeabilized with 2 mg/mL hyaluronidase (Sigma) for 1 h at 37°C and put in 4%PFA/PBS for 20 min at RT for post‐fixation. The embryos were blocked for 4 h with blocking solution (10% donkey serum and 10% goat serum in 0.1% PBST) at RT and incubated for 2 days with primary antibody solution at 4°C (mouse anti‐Desmin, Invitrogen MA5‐13259, dilution 1:250; rabbit anti‐Sox9, Sigma HPA001758, dilution 1:150; rabbit anti‐GFP, ABCAM, AB290, dilution 1:500).

After washing in 1× PBS, the embryos were incubated with secondary antibodies (donkey anti‐mouse AF568 IgG (H + L), Invitrogen A10037, dilution 1:500, or donkey anti‐rabbit AF568 IgG (H + L), Life technologies A10042, dilution 1:1000; goat anti‐rabbit AF488 IgG (H + L) Invitrogen A1108, dilution 1:750) in 1% BSA in 0.1% PBST overnight and washed again in 1× PBS. Finally, the embryos were again fixed with 4% paraformaldehyde in 1× PBS buffer solution and stored at 4°C. Specimens were examined and photographed using a Leica MZ16F stereomicroscope.

To visualize individual abdominal muscle fibers and to investigate the fusion of the muscle fibers, selected stained embryos were embedded in Gelatin–BSA–Sucrose (GBS) mixture (0.49% Gelatine, 30% BSA, 20% Sucrose), sectioned with a vibratome (Leica VT 1000S) at 100 μm and mounted in Prolong Diamond (Invitrogen).

### 2.5 Electroporation

For electroporation of the somites, we incubated fertilized chicken eggs for 60–65 h, until they reached HH‐stage 14–17.

We prepared the eggs following in principle the technique described by Scaal et al. (2004).

The upper side of the eggshell was stabilized by broad adhesive tape. After withdrawing 3–4 mL of albumen, a window was cut into the eggshell with scissors.

To visualize the embryo, we injected India ink (Rotring, diluted 1:10 with 1× PBS‐Buffer) into the yolk below the embryo.

Injection needles made from borosilicate glass capillaries (O.D. 1.5 mm; I.D. 1.10 mm; Science Products GmbH) were drawn on a Sutter P‐97 Puller.

The injection of the somites 24–30 with muscle‐specific markers was done individually for each somite in a lateral to medial direction, using 1 μg/μL plasmid solution.

The cathode (−) was placed under the embryo and the anode (+) on top of the embryo in an oblique plane, with the cathode on the left and the anode on the right of the embryo (both platinum wire Ø 0.4 mm). We applied 3 pulses at 20 Volt, 20 ms length, 50 ms space with a NEPA21 Type II Super Electroporator (Nepagene).

The injected embryos were covered with 1–1.5 mL 1× PBS, the egg window was closed with adhesive tape and eggs reincubated until they reached the desired stage.

Embryos at HH‐stages 24–30 were isolated, prepared as described above and fixed with 4% paraformaldehyde. Specimens were examined and photographed using a Leica MZ16F stereomicroscope. Where applicable, after the electroporation, embryos were additionally subject to immunohistochemistry as described above.

### 2.6 Constructs used

The constructs used for electroporation are muscle fiber‐specific plasmids driving the expression of a membrane‐targeted eGFP (Tol2‐MLC:eGFP‐CAAX), here abbreviated as MLC‐GFP, and membrane‐targeted dTomato (Tol2‐MLC:mbdTomato), here abbreviated as MLC‐Tomato, together with CAGGS‐Transposase for stable integration (Toulouse et al., 2025). All plasmids were kindly provided by Christophe Marcelle, University of Lyon, France.

### 2.7 Imaging

Whole‐mount specimens were examined and photographed using a Leica MZ16F stereomicroscope. Transversal slices were imaged with a Leica THUNDER Imager 3D Tissue or a Zeiss LSM880 Airyscan confocal microscope.

## 3 RESULTS

### 3.1 Chicken abdominal muscle anatomy

**FIGURE 2:**
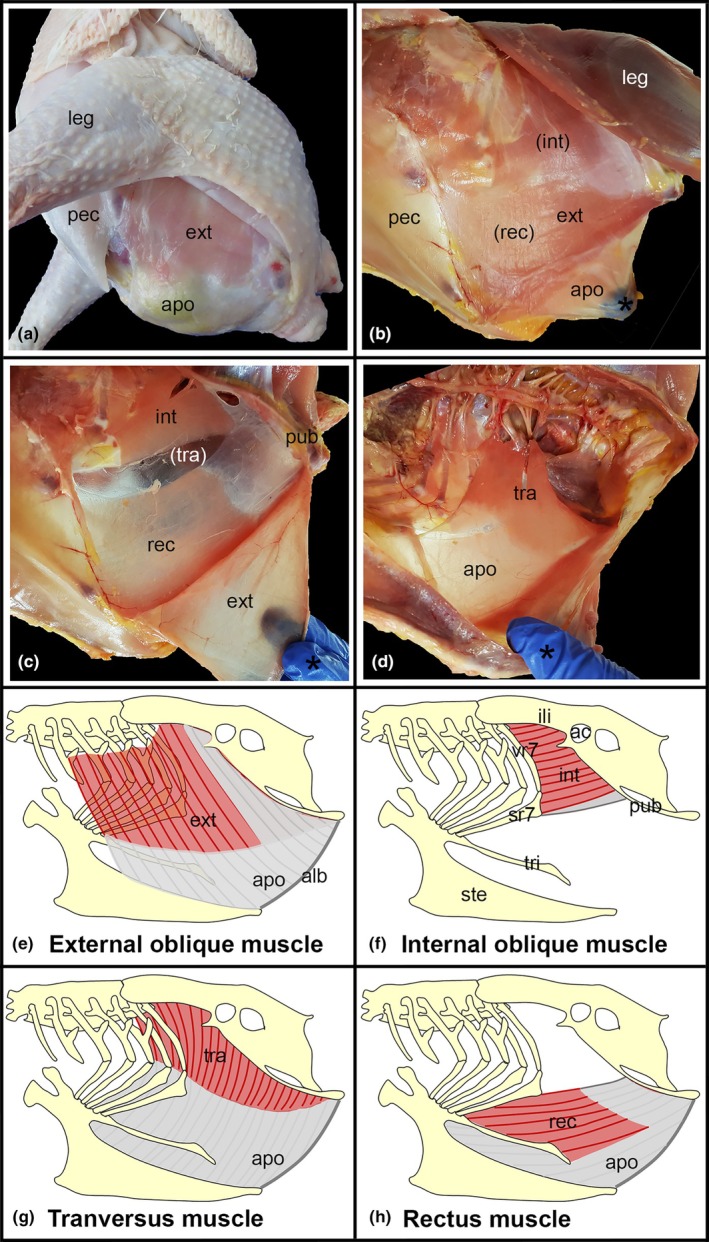
Anatomy of abdominal muscles. (a–d) Dissection of an adult specimen. (e–h) Anatomy of individual abdominal muscles in the adult chicken based on data from the literature and own dissections, lateral view, dorsal to the top, and cranial to the left. (a) Overview of a chicken before evisceration. The skin on the ventral side has been removed to show the superficial layer of the abdominal wall with the external oblique muscle and the ventromedial *Aponeurosis abdominis*. (b) Lateral view of the superficial layer of the abdominal wall after complete removal of the skin and evisceration. The left leg has been maximally abducted to expose as much of the abdominal muscles as possible. Note that the external oblique muscle is so delicate, especially in its caudal aponeurotic part, to be sufficiently transparent to allow the underlying internal oblique and rectus muscles to become visible. (c) Lateral view. The external oblique muscle has been cut along its dorsal origin and folded out lateroventrally to expose the underlying internal oblique and rectus muscles, which are partially connected via a delicate aponeurosis underneath which the transversus muscle is slightly shining through. (d) Medial view. After removal of the intestines and the peritoneum the transversus muscle and its ventral aponeurosis are seen as the innermost layer of the abdominal body wall. (e) Schematic drawing of the external oblique muscle, *M. obliquus externus abdominis*. (f) Schematic drawing of the internal oblique muscle, *M. obliquus internus abdominis*. (g) Schematic drawing of the transversus muscle, *M. transversus abdominis*. (h) Schematic drawing of the rectus muscle, *M. rectus abdominis*. ac, *Acetabulum*; alb, *Linea alba*; apo, *Aponeurosis abdominalis*; ext., *M. obliquus externus abdominis*; ili, preacetabular part of *Os ilium*; int., *M. obliquus internus abdominis*; pec, *M. pectoralis*; pub, *Os pubis*; rec, *M. rectus abdominis*; sr7, seventh sternal rib; ste, *Corpus sterni*; tra, *M* *. transversus abdominis*; tri, *Trabecula intermedia sterni*. vr7, seventh vertebral rib; asterisk*, gloved finger positioning the specimen.

Despite the importance of the chicken embryo as a model to study muscle development, the anatomy of the abdominal musculature is not well described. We therefore combined a survey of the available literature (Baumel, 1993; Engert, 1900; Gadow, 1891; Ghetie, 1976; König & Liebich, 2001; Schummer, 1973) and the results of our own dissections of adult chicken to revisit chicken abdominal muscle anatomy. We visualized abdominal muscle morphology both in dissected specimens and in semi‐schematic drawings in order to provide a basis to correctly identify the emerging muscle anlagen during our observations on abdominal muscle morphogenesis (Figure 2).

Similar to mammalian anatomy, the chicken abdominal wall consists of three layers of lateral muscles including the external oblique, internal oblique and transversus muscles, and the rectus muscle covering the lateroventral abdominal wall, whereas the medioventral abdominal wall consists of a broad aponeurosis (*Aponeurosis abdominis*) connecting the bilateral muscles over the midline. The anatomy of the individual abdominal muscles can be described as follows (Figure 2):

The external oblique muscle envelops the abdomen on its outer surface. It originates in its cranial part from the uncinate processes and lateral surface of a variable number of vertebral ribs, and in its caudal part from the ventral margin of the ilium and the pubis. The latter lumbal portion of the muscle is mainly aponeurotic. The muscle fibers run in a ventrocaudal direction and insert via a delicate aponeurosis in its cranial part at the lateral margin of the *Trabecula mediana scapulae* and the lateral margin of the *Corpus sterni*, and in its caudal part at the *Aponeurosis abdominis*, where it unites with the aponeurosis of the contralateral counterpart in the ventral midline, the *Linea alba*.

The internal oblique muscle represents the intermediate layer of the lateral abdominal wall and consists of muscle fibers which originate at the ventral border of the preaceteabular ilium and the pubis and run in a cranial direction to insert at the last vertebral rib. At its costal insertion and along its ventral margin, it is connected to the rectus muscle via a thin aponeurosis.

The transverse abdominal muscle represents the inner layer of the abdominal wall, which is immediately covering the peritoneal cavity. Its fibers run in dorsoventral, that is, transverse direction, originating in its cranial part from the inner surface of the last three ribs (5th, 6th, and 7th rib), and in its caudal part from the ventral margin of the preacetabular ilium and the pubis. The ventral part of the muscle is aponeurotic and inserts, like the external oblique, at the lateral margin of the sternum and forms the inner part of the *Aponeurosis abdominis*, before it meets its contralateral counterpart in the *Linea alba*.

The rectus abdominis muscle originates from the lateral margin of the *Corpus sterni*, the *Trabecula intermedia sterni*, and the caudalmost sternal rib, and runs caudally to insert at the caudal part of the pubis. Both original and insertional ends of the muscle are partly aponeurotic, and a thin aponeurosis at its dorsal margin connects it to the internus muscle.

### 3.2 Abdominal muscle morphogenesis

**FIGURE 3:**
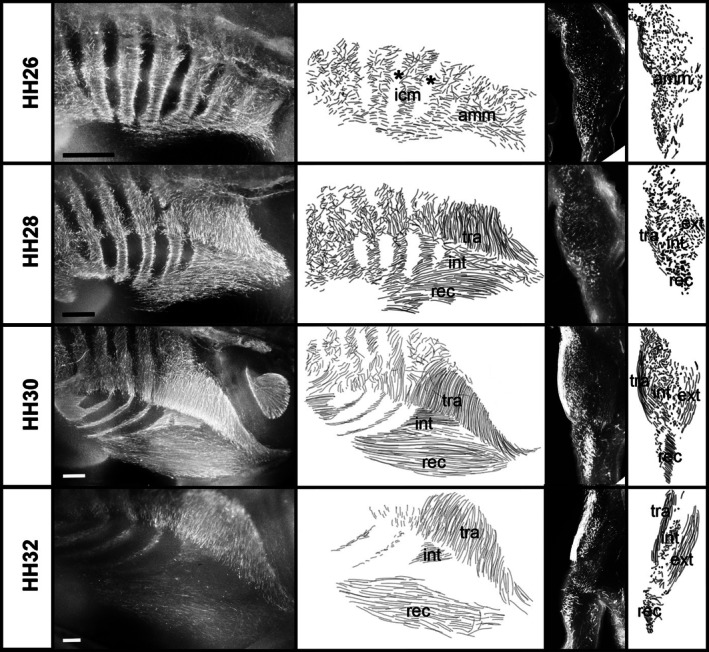
Morphogenesis of abdominal muscles from HH‐stage 26 to HH‐stage 32 revealed by immunohistochemistry using an antibody against the muscle‐specific intermediate filament desmin. Whole mounts (left column) in medial view, cranial is to the left; in the transverse sections (right column), medial is to the left. Whole mounts and sections are supplemented by semi‐schematic graphics for better visualization of individual muscles. Note the progressive sculpting of the definitive anatomical muscles from a common abdominal muscle mass by changes in muscle fiber orientation and interjacent connective tissue (unstained). The external oblique muscle is not visible in whole mounts in medial view, but only in sections. amm, abdominal muscle mass; ext, *M. obliquus externus abdominis*; icm, intercostal muscle; int, *M. obliquus internus abdominis*; rec, *M. rectus abdominis*; tra, *M. transversus abdominis*; asterisk*, non‐stained rib anlagen. Scale bars: 500 μm.

Previous descriptions of abdominal muscle development in the chicken embryo had been limited to transverse sections and to non‐muscle‐specific staining methods (Christ et al., 1983; Engert, 1900). In order to get a more detailed insight into abdominal muscle formation, we subjected whole‐mount embryos to immunohistochemistry using a muscle‐specific anti‐desmin antibody. As the internal oblique, transverse, and rectus muscles are best visible in medial view, embryos were beforehand cut in half along the midline and carefully eviscerated. The external oblique muscle is not sufficiently visible in whole mounts, neither in medial nor in lateral view, as it is obscured by adjacent muscles and connective tissue. Therefore, after studying the abdominal musculature in medial view, the embryo halves were transversely sectioned with a vibratome, thus revealing all muscle sheets including the external oblique muscle. Whole‐mount preparations and sections together allowed muscle morphology to be studied in three dimensions (Figure 3).

At HH‐stages 24 and 25 (Embryonic day E4.5–5), the prospective abdominal musculature is still segmental, consisting of ventrolaterally extending hypaxial myotomes with craniocaudally oriented parallel myocytes in each segment. At this stage, the abdominal muscle anlagen are reminiscent of intercostal muscle anlagen (data not shown, see Khabyuk et al., 2022).

At HH‐stage 26 (E5), the segment borders caudal to the last rib blur as the hypaxial myotomal cells start to elongate beyond segment borders, forming a common abdominal muscle mass. In addition, in the ventralmost part of the intercostal muscles of the 4th to 7th ribs, the muscle fibers lying distal to the tips of the ribs also cross segments and form a population of craniocaudally arranged fibers. Moreover, some of the muscle fibers in the proximal part of the lumbar, that is, post‐costal, segments as well as of the intercostal muscles lose their craniocaudal, parallel orientation and turn towards a dorsoventral orientation. This is most prominently seen in the medialmost layer of the myotomes, but also in some cells at the lateral margin.

At HH‐stage 28 (E5.5–6), the muscle fibers of the proximal (dorsal) half of the post‐costal musculature, which were at random orientation at HH‐stage 26, are stabilized in a dorsoventral orientation and elongate in parallel to form a curtain‐like muscle sheet with perpendicular muscle fibers. Similarly, the previously random muscle fibers on the medial surface of the intercostal spaces of the ribs increasingly arrange in dorsoventral orientation. In both locations, namely the medialmost fibers are concerned, thus forming the anlage of the transversus muscle. At the same time, the craniocaudally extending muscle fibers distal to the tips of the 5th to 7th rib unite with the ventralmost post‐costal muscle fibers, both by considerable elongation, and form a common ventral muscle mass which is the anlage of the rectus abdominis muscle. The internus muscle, which is formed by fibers dorsal to the rectus and lateral to the transversus, as well as the externus muscle, is only vaguely visible as slightly oblique, craniocaudal fibers at this stage.

In HH‐stage 30 (E6.5–7), both the rectus abdominis and transversus abdominis muscles show a massive increase of the number and density of parallel muscle fibers and have further taken shape. The transversus muscle extends from the ventral part of the ilium and the pubis, as well as from the caudalmost 2–3 vertebral ribs, in ventral direction to cover the dorsal aspect of the lateral abdominal wall. Note that the aponeurotic ventral portion of the muscle is not visible in these desmin stainings. Between transversus and external oblique muscles, the internal oblique muscle becomes clearly visible as a sheet of parallel muscle fibers extending craniocaudally in a slight dorsocaudal to ventrocranial angle, and attaching to the caudalmost vertebral rib. Ventral to the internal oblique, the rectus muscle is clearly visible extending from the last sternal rib and the sternum to the pubis. The external oblique appears in sections as a well‐formed package of oblique muscle fibers which run in ventrocaudal orientation. Thus, at HH‐stage 30, all abdominal muscles are distinguishable, even though the individual muscle sheets of the lateral abdominal muscles are not yet fully separated, as is visible in transverse sections. This stage was used in the cell tracking experiments in the following.

In HH‐stage 32 (E7.5), all intermuscular spaces are clearly visible in sections, so that all abdominal muscles are individually separated from each other and have reached their definitive anatomical position in the body wall. The intermuscular spaces are likely to be filled with non‐stained, somatopleura‐derived mesenchyme or connective tissue. Note that the perpendicular fibers of the transversus muscle are only visible in the dorsolateral aspect of the abdominal wall, as the ventromedial aspect of the muscle is a thin aponeurosis which is not stained by the muscle‐specific antibody used here.

### 3.3 Contribution of individual somites to abdominal muscles

It has been unequivocally shown that the abdominal musculature in birds is formed by the hypaxial portion of the somites (Christ et al., 1983). However, as to the segments participating in abdominal muscle formation, the literature is controversial (Chevallier, 1979; Christ et al., 1983; Rees et al., 2003; Seno, 1961).

**FIGURE 4:**
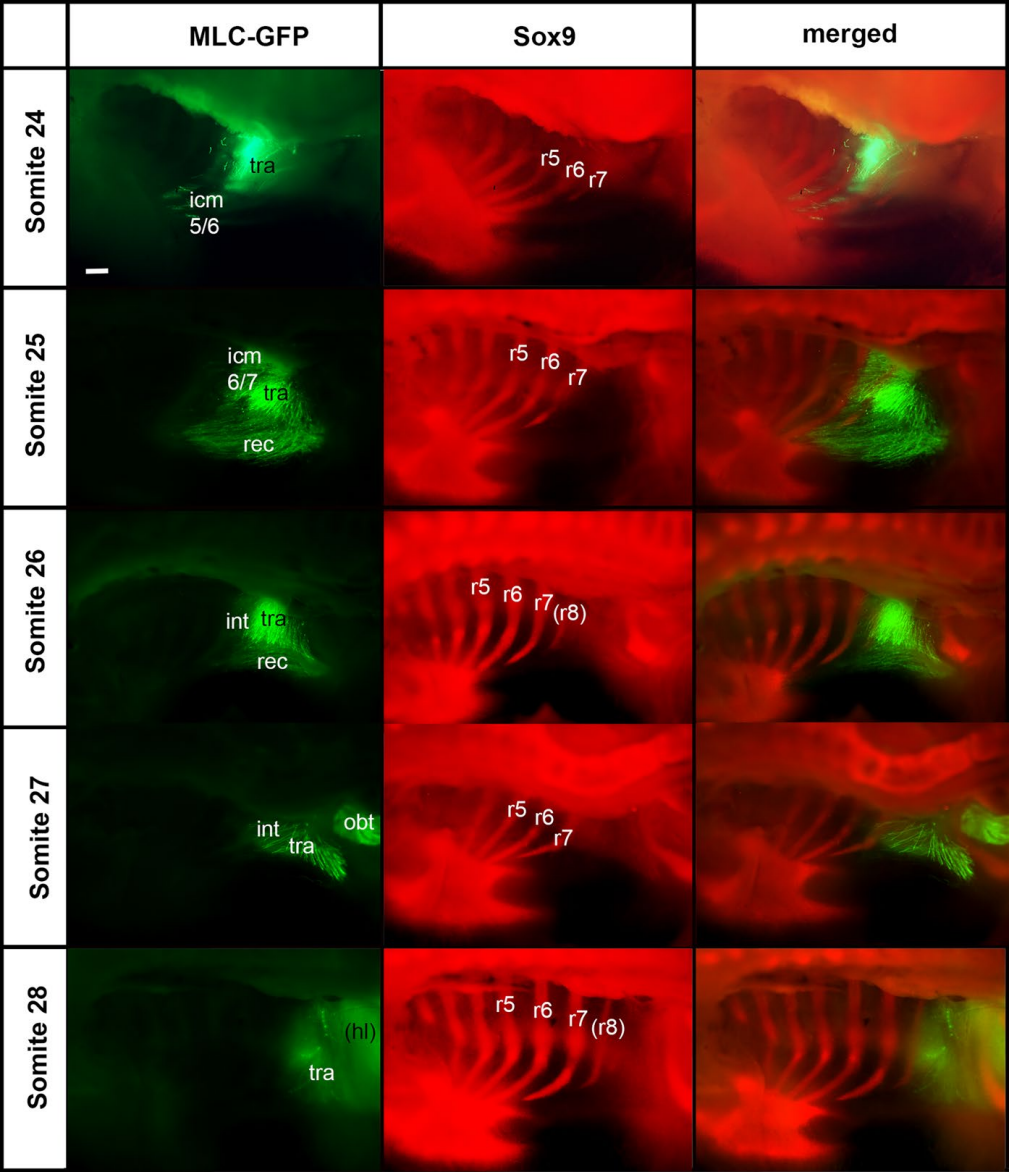
Contribution of individual somites to abdominal muscles correlated with the thoracic skeleton. Whole‐mount embryos of HH‐stage 30, medial view, cranial to the left. Single somites have been electroporated with a plasmid coding for a muscle‐specific GFP reporter construct (MLC‐GFP), which has been visualized by immunohistochemistry using an antibody against GFP (green color). Embryos have been additionally stained with an antibody against the cartilage marker Sox9 (red color) to show the position of the stained muscles relative to the ribs and pelvic girdle. Note that at the stages examined the 7th rib is only partly formed and does not yet reach the sternum (Khabyuk et al., 2022). Muscle cells from somite 24 are seen in the intercostal muscles between the 5th and 6th rib, with some fibers extending into the cranially neighboring intercostal space, and in fibers of the transversus muscle. Fibers of the internal oblique muscle are hardly visible here as they are covered by the transversus fibers. Muscle cells from somite 25 are found in the intercostal space between the 6th and 7th rib, and in all abdominal muscles. Muscle cells from somite 26 are found in the intercostal space between the 7th and 8th rib, and in all abdominal muscles. Muscle cells from somite 27 are seen in the internal oblique and transversus muscle, as well as in the obturatorius muscle of the hindlimb. Muscle cells from somite 28 are seen in the caudalmost part of the transversus muscle and in hindlimb musculature. Note that the external oblique muscle is not visible in these medial views. (hl), non‐identified hindlimb muscles; icm5/6, intercostal muscle between 5th and 6th rib; icm6/7, intercostal muscle between 6th and 7th rib; int, *M. obliquus internus abdominis*; obt, *M. obturatorius* (a hindlimb muscle); r5‐r7, 5th to 7th rib; (r8), inconstant 8th rib; rec, *M. rectus abdominis*; tra, *M. transversus abdominis*. Scale bar: 500 μm.

We reexamined this question by labelling individual somites by electroporation with a muscle‐specific GFP expression vector, MLC‐GFP, at HH‐stage 14–17 and monitoring the presence of the MLC‐GFP marker after 5 days of re‐incubation in the abdominal muscles of embryos at approximately HH‐stage 30. In order to acquire these data, it was vital to exactly identify the segment number during electroporation. This is difficult at the stages used because the more cranial somites are hard to discern due to overlying tissue and beginning rotation of the embryo (Burke et al., 1995; see also Section 2). To validate our counting, we performed double immunohistochemistry with antibodies against MLC‐GFP and the cartilage marker Sox9. Based on the segmental identity map by Burke et al. (1995), we defined the relevant segments as follows: Somite 24 gives rise to intercostal muscles between ribs 5 and 6, Somite 25 gives rise to intercostal muscles between ribs 6 and 7, Somite 26 gives rise to intercostal muscles between ribs 7 and the temporary 8th rib if present, or else only to muscles caudal to the 7th rib (Figure 4; see also Figure 1). Somites 27 and beyond could not be defined this way but were extrapolated based on the warranted accuracy of the previous counts.

**FIGURE 5:**
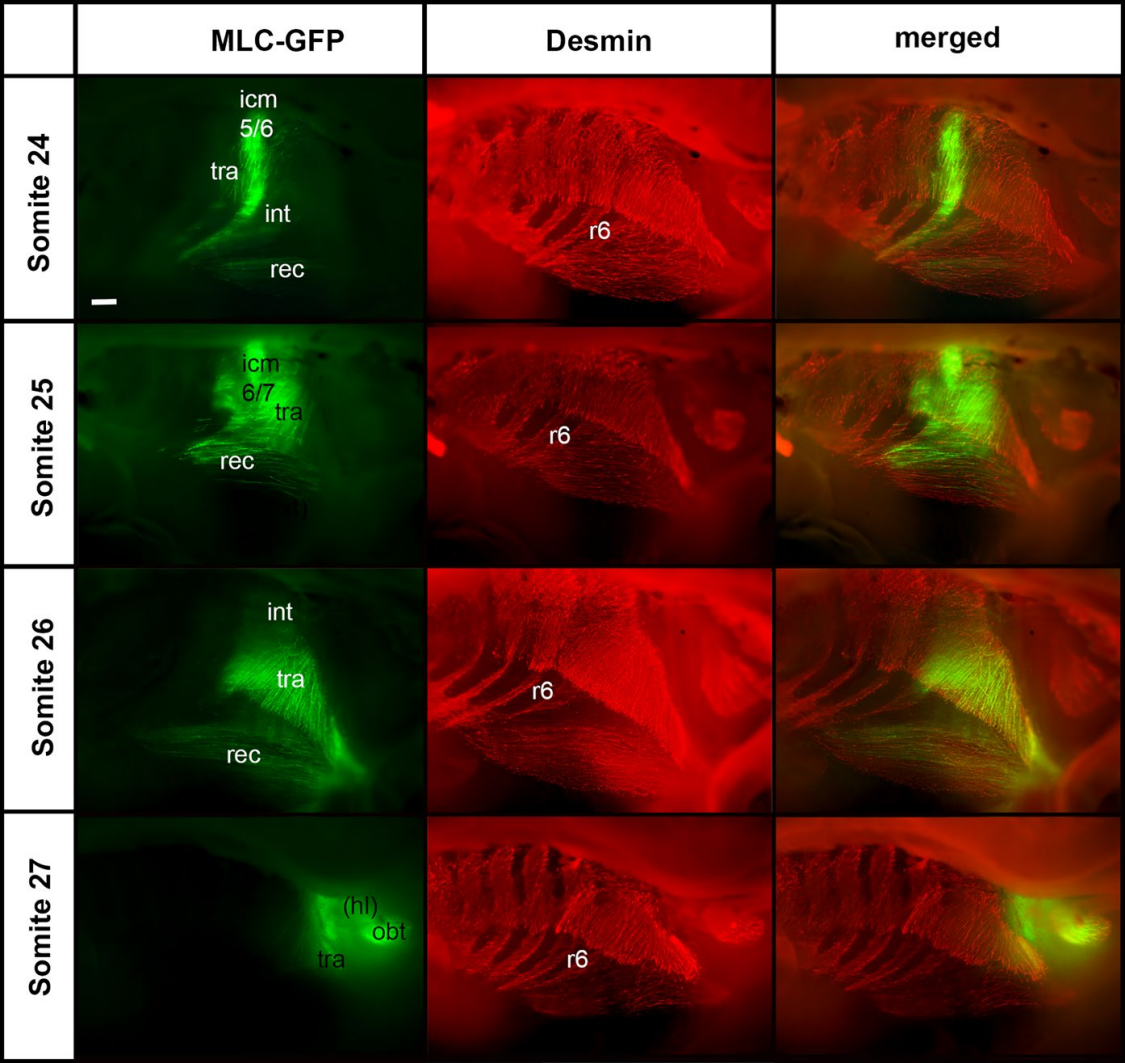
Contribution of individual somites to abdominal muscles correlated with total muscle staining. Whole‐mount embryos of HH‐stage 30, medial view, cranial to the left. Single somites have been electroporated with a plasmid coding for a muscle‐specific GFP reporter construct (MLC‐GFP), which has been visualized by immunohistochemistry using an antibody against GFP (green color). Embryos have been additionally stained with an antibody against the muscle‐specific intermediate filament desmin (red color) to show all skeletal muscles, thus allowing to determine which part of the anatomical muscles derive from the respective segment. Muscle cells from somite 24 are seen in the intercostal space 5/6, in the cranial part of the transversus muscle, and with few fibers in the internal oblique and rectus muscles. Muscle cells from somite 25 are seen in the intercostal space 6/7, in the intermediate part of the transversus muscle, in few fibers of the internal oblique, and in the rectus muscle. Muscle cells from somite 26 are seen in all abdominal muscles. Muscle cells from somite 27 are seen in the caudal part of the transversus muscle and in hindlimb musculature. Note that the external oblique muscle is not visible in these medial views. (hl), non‐identified hindlimb muscles; icm5/6, intercostal muscle between 5th and 6th rib; icm6/7, intercostal muscle between 6th and 7th rib; int, *M. obliquus internus abdominis*; (ext), *M. externus abdominis* weakly discernible behind the internal oblique and rectus muscles; obt, *M. obturatorius* (a hindlimb muscle); r6, 6th rib annotated for orientation; rec, *M. rectus abdominis*; tra, *M. transversus abdominis*. Scale bar: 500 μm.

Having thus validated our counting method, we double‐stained embryos electroporated with MLC‐GFP with antibodies against GFP and the muscle marker Desmin. In this experiment, Desmin stained all muscle cells in the embryo, whereas GFP only stained muscle cells of the electroporated segment. This enabled us to determine which muscles, or part of muscles, receive muscle cells of the respective segmental origin (Figure 5).

**FIGURE 6:**
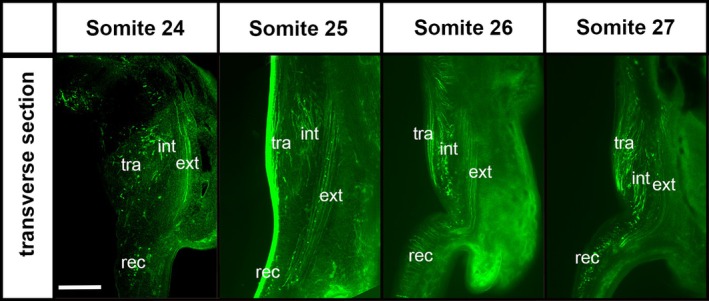
Contribution of individual somites to abdominal muscles in transverse section. Whole‐mount embryos of HH‐stage 30. Single somites have been electroporated with a plasmid coding for a muscle‐specific GFP reporter construct (MLC‐GFP), which has been visualized by immunohistochemistry using an antibody against GFP (green color). In the specimens shown, somites 24–27 all give rise to all muscles of the abdominal wall including the external oblique muscle: ext, *M. obliquus externus abdominis*; int, *M. obliquus internus abdominis*; rec, *M. rectus abdominis*; tra, *M. transversus abdominis*. Scale bar: 250 μm.

The external oblique muscle is neither visible in the whole‐mount embryos in medial view as it is covered by other muscles and connective tissue layers, nor in lateral view due to thick dermal tissue and massive leg musculature. We therefore made transverse sections of embryos electroporated with MLC‐GFP and stained with an antibody against GFP, thus showing all abdominal muscle layers receiving muscle cells from the respective segments including the external oblique muscle (Figure 6). Our results of these experiments together were as follows:

**Somite 24** predominantly gives rise to intercostal muscles between the 5th and 6th rib, but also gives rise to fibers of the cranial part of the transversus muscle overlying this intercostal space. Moreover, it gives rise to a minor number of fibers of the rectus abdominis, and some fibers at the rectus–internus interface which are difficult to securely attribute to either of these muscles. Sections revealed that somite 24 also gives rise to the external oblique muscle. Interestingly, we consistently found fibers not only in the strongly labelled intercostal space between the 5th and 6th rib, but also a few fibers in the cranially neighboring intercostal space between the 4th and 5th rib (see Figures 4 and 5). As it is unlikely that one somite contributes to two neighboring intercostal muscles, we speculate that this observation is either due to segment‐crossing fibers extending cranially into the next segment, and/or to labelled fibers from the external oblique muscle shining through the intercostal space from the lateral surface of the specimen.

**Somite 25** gives rise to intercostal muscles between the 6th and 7th rib, as well as to the intermediate part of the transversus. It also provides fibers which cover the 7th rib on its medial side and extend in craniocaudal orientation to the caudal margin of the 6th rib, which we consider as part of the anlage of the internal oblique muscle. It is to be noted that this is at variance with the adult morphology, where the internal oblique muscle is known to be attached to the caudalmost rib, that is, the 7th rib, which at that stage is not yet fully formed as it has not reached contact with the sternum (Khabyuk et al., 2022). Moreover, somite 25 strongly participates in the formation of the external oblique muscle and to fibers of the rectus abdominis.

**Somite 26** gives rise to the largest part of the transversus muscle, covering a large craniocaudal extent of the transversus from the 6th rib to its caudal end, and to most fibers of the rectus muscle. It also gives rise to the bulk of the internal and external oblique muscles. In cases where a temporary 8th rib is formed, the muscles between the 7th and 8th rib are also derived from this segment (see Figure 4). Thus, somite 26, together with somite 25, can be considered as the main source of abdominal muscles in the chicken embryo.

**Somite 27** provides fibers to the transversus muscle, most of them to the caudalmost part and only a few fibers reaching also the intermediate part. The amount of contribution of this segment to the abdominal musculature appears quite variable; in some but not all specimens, it also contributes individual fibers to the internal and external oblique muscles (see Figures 4 and 6 in contrast to Figure 5). Apart from the abdominal muscles, we found many muscle cells from somite 27 also in the obturatorius muscle, which belongs to the hindlimb musculature that is well visible in the obturate foramen in our medial preparations (see Figures 4 and 5).

**FIGURE 7:**
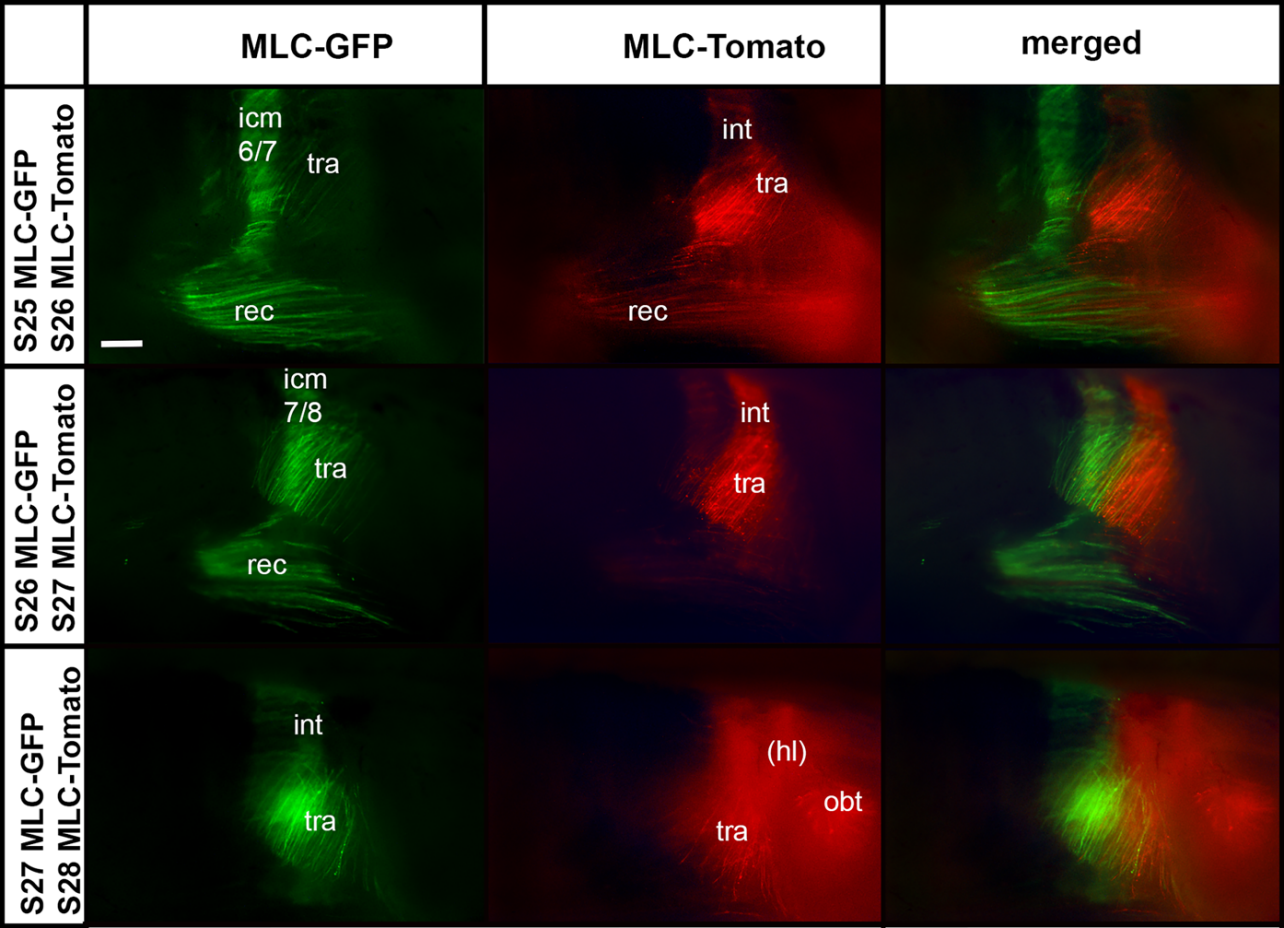
Proportionate contribution of two neighboring somites to abdominal muscles. Thoraco‐lumbal transition zone of whole‐mount embryos of HH‐stage 30, medial view, cranial to the left. Two neighboring somites (somites 25 and 26, 26 and 27, or 27 and 28) have been electroporated, the cranial neighbor with a plasmid coding for a muscle‐specific GFP reporter construct (MLC‐GFP) and the caudal neighbor with a plasmid coding for a muscle‐specific Tomato reporter construct (MLC‐Tomato). In the transversus muscle, the dorsoventrally oriented muscle fibers approximately maintain their segmental arrangement along the craniocaudal extent of the muscle, with muscle cells from more cranial somites contributing to fibers of more cranial muscle parts. By contrast, the craniocaudally oriented fibers of the rectus muscle mix randomly so that segment borders are obliterated. (hl), non‐identified hindlimb muscles; icm6/7, intercostal muscle between 6th and 7th rib; icm7/8, intercostal muscle between 7th and variant 8th rib; int, *M. obliquus internus abdominis*; obt, *M. obturatorius* (a hindlimb muscle); rec, *M. rectus abdominis*; tra, *M. transversus abdominis*. Scale bar: 500 μm.

**Somite 28** mainly forms hindlimb muscles but contributes a few fibers to the caudalmost part of the transversus muscle (see Figures 4 and 7).

**Somite 29** does not participate in abdominal muscle formation according to our experiments (data not shown).

To sum up these experiments, we found that somites 24–27 all contribute to all abdominal muscles; however, at variable extent and with considerable individual variation, that somites 25 and 26 provide the largest amount of abdominal muscle cells in comparison with other segments, and that somite 28 provides a minor contribution to the caudal part of the transversus muscle only.

### 3.4 Heterogeneous segmental organization of abdominal muscles

All trunk muscles of the ventrolateral body wall derive from the hypaxial myotomes of the somites and are thus primarily segmental (Christ et al., 1983). In contrast to intercostal muscles, which overtly maintain their segmental organization to adulthood, the abdominal muscles form wide muscle sheets without morphologically visible segmental characteristics. Our single somite electroporations reported above have indicated that at least in the transversus muscle, the segmental origin of the muscle fibers is maintained in the muscle sheets with respect to the origin of different parts of the muscles. In order to examine this in more detail, we electroporated two neighboring somites alternately with different expression constructs, MLC‐GFP and MLC‐Tomato, and monitored their derivatives in the abdominal musculature (Figure 7). We found that in the transversus muscle, somites 25 to 27 contribute to different parts of the transversus muscle in craniocaudal sequence, with somite 25 giving rise to more cranial fibers than somite 26, and somite 26 giving rise to more cranial fibers than somite 27. Thus, the transversus muscle approximately maintains its segmental origin along its craniocaudal extent. However, this segmental organization is not strict, as fibers from each segment overlap with those of neighboring segments. By contrast, in the rectus abdominis muscle, we did not find a segmental arrangement of fibers of different segmental origin, as fibers of both segments appeared randomly mixed within the muscle. For the internal oblique and external oblique muscles, which are not or only partially visible in our approach, we were unable to achieve precise results. To conclude, the segmental organization of the abdominal musculature is heterogeneous, with the transversus muscle roughly maintaining segmental organization along the craniocaudal axis, and the rectus abdominis muscle containing mixed fibers of different origin with no detectable segmental pattern.

### 3.5 Mixing but no fusion of muscle fibers of different segmental origin

**FIGURE 8:**
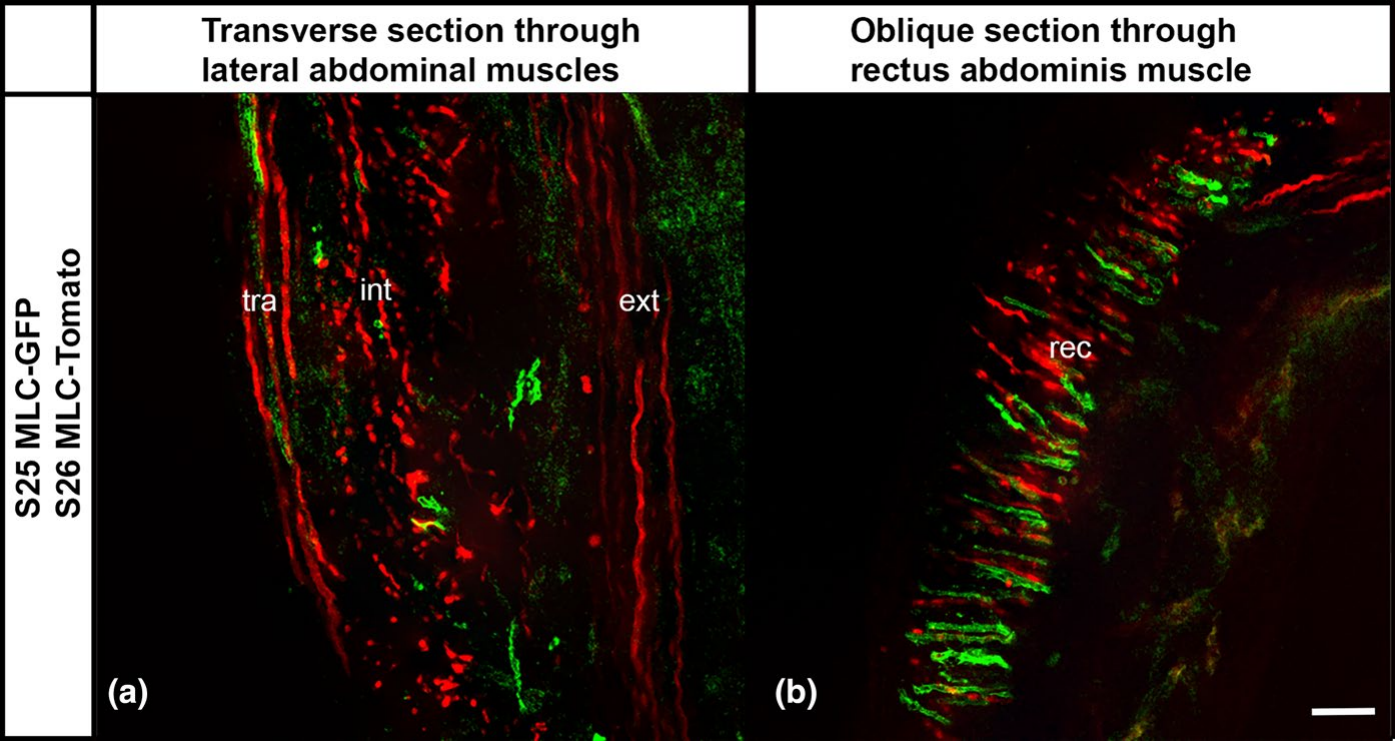
Relation of muscle fibers originating from neighboring somites on cellular level. Transverse or oblique section through the lateral abdominal muscles and the rectus muscle, respectively. Two neighboring somites (somites 25 and 26) have been electroporated, the cranial neighbor with a plasmid coding for a muscle‐specific GFP reporter construct (MLC‐GFP) and the caudal neighbor with a plasmid coding for a muscle‐specific Tomato reporter construct (MLC‐Tomato). Whole‐mount embryos have been sectioned and analyzed under a confocal microscope. Note that in both cases, cells of different segmental origin mix to various extent but never fuse to form muscle fibers of mixed segmental origin. int, *M. obliquus internus abdominis*; rec, *M. rectus abdominis*; tra, *M. transversus abdominis*. Scale bar: 50 μm.

As detailed above we have shown that in abdominal muscles, fibers of different segmental origin intermingle, either partly as in the transversus muscle, or largely as in the rectus abdominis muscle. During embryonic muscle growth, muscle fibers elongate by fusion of primary myotomal cells and/or resident muscle precursor cells (Sieiro‐Mosti et al., 2014). One possibility is that during this fusion‐driven muscle fiber formation, the segmental identity of individual fibers is maintained, in that fusion occurs only between cells of the same somitic origin. The other possibility is that there is also fusion of muscle precursor cells originating from different somites, resulting in abdominal muscle fibers of mixed segmental origin. To determine this, we again electroporated neighboring somites alternately with MLC‐GFP and MLC‐Tomato, and examined the muscle fibers in vibratome sections of the abdominal wall at high magnification under a confocal microscope (Figure 8). We found in all abdominal muscles exclusively individual muscle fibers with either green or red fluorescence, thus originating from different somites. We never detected muscle fibers showing both, MLC‐GFP and MLC‐Tomato expression, in the same muscle fiber. We therefore conclude that during abdominal muscle formation, cells of different segmental origin intermingle but do not fuse. If this is a general principle in the formation of large muscles or specific for abdominal muscles remains to be determined.

## 4 DISCUSSION

### 4.1 Abdominal muscle morphology

The anatomy of avian abdominal musculature is not well described in the literature. To provide a morphological basis for the interpretation of our studies on embryonic abdominal muscle formation, we dissected the abdominal wall of the adult chicken to study the specific topography of the chicken abdominal muscles. These results, in combination with a survey of the previous literature, allowed us to make graphic representations of the individual abdominal muscles, which have not been available in the literature so far. This enabled us to safely identify the individual muscle anlagen during abdominal wall morphogenesis and will be the foundation of further studies in this field.

In amniotes, the ventral body wall is archetypically characterized by ribs interconnected by two muscular layers, external and internal intercostal muscles. However, this ancestral amniote bauplan is only preserved in a few reptilian taxa like *Sphenodon* or snakes, which indeed show rib formation in all truncal segments (Starck, 1979). The majority of amniotes, including all birds and mammals, show a severe reduction of the rib anlagen at lumbosacral levels, with only rudimentary ribs persisting as costal processes and as integrated parts of the sacral skeleton, thus dividing the trunk in a rib‐bearing thoracic and a rib‐less abdominal region. In the absence of bony insertions at the ribs, the intercostal muscles at lumbosacral levels elongate to large muscle sheets, in which the orientation of the muscle fibers of the external and internal intercostals is reflected in the corresponding orientation of the external and internal oblique muscles, respectively. In addition, and less evident to correlate with intercostal muscle anlagen, the transversus and rectus muscles are formed. Thus, the abdominal ventrolateral body wall is a non‐skeletal, musculofibrous sheath, which consists mainly of the three‐layered complex of external oblique, internal oblique, and transverse abdominal muscles, as well as the ventral rectus abdominis muscle, and their corresponding aponeuroses.

In the chicken, this basic arrangement of abdominal muscles is similar to the situation in mammals and humans, so that the chicken embryo is a well‐suited model system to study ventral body wall development with a clinical perspective. At variance with the mammalian situation, birds do not have a *bona fide* rectus sheath, but instead a broad abdominal aponeurosis as a ventromedial extension of the transversus and external oblique muscles without contribution of the internal oblique muscle. The internal oblique muscle does not contribute to the abdominal aponeurosis but is restricted to the lateral aspect of the abdominal wall where it inserts at the caudalmost vertebral rib and has a delicate aponeurotic connection to the rectus muscle. The rectus muscle thus appears as the ventral continuation of the internal oblique muscle, with fibers in similar, craniocaudal orientation but inserting at the sternal rib and the sternum. In contrast to the well‐known situation in humans, the avian rectus muscle does not have tendinous intersections. These differences between avian and mammalian anatomy of the abdominal muscles should be kept in mind when interpreting data from avian model systems.

### 4.2 Abdominal muscle morphogenesis

In order to monitor the development of the abdominal muscles, we performed immunohistochemistry using a muscle‐specific anti‐desmin antibody on embryos of different developmental stages. We documented the formation of the individual abdominal muscles as they arise from the initially uniform abdominal muscle mass in whole‐mount embryos in combination with sections, to provide a three‐dimensional pattern of abdominal muscle morphogenesis. We observed substantial muscle splitting at HH‐stage 28, and the final arrangement of muscles with distinct intermuscular tissue and aponeurotic anlagen is reached between HH‐stages 30 and 32.

This is consistent with two earlier studies based exclusively on transverse sections. Christ et al. (1983) monitored the timing and morphogenesis of the abdominal musculature by quail‐chick transplantation experiments. They found that the ventrolateral lips of the dermomyotomes, while forming the hypaxial myotomes, invade the somatopleura at embryonic day 4 (E4), which corresponds to HH‐stages 23–25. Within the hypaxial myotomes, extracellular spaces appear which are filled by mesenchymal connective tissue precursor cells from the somatopleura and which split the myotome in several muscle layers. Ultrastructural examinations of the same group indicated that the muscle splitting occurs at E6, which corresponds to late HH‐stage 28, and later stages (Jacob et al., 1981). At E7 (HH‐stages 30–33), they found all abdominal muscle anlagen distinguishable with intermuscular septa and regularly oriented myofibers, and at E10 (HH‐stage 36), the final arrangement and position of the muscles of the abdominal wall is reached (Christ et al., 1983). The pioneering work by Engert (1900) came to similar results, even though with slightly different timing. According to Engert, the ventrolateral lip of the dermomyotomes at interlimb level persists until E4 but dissolves at E5 into a compact cell mass, which does not yet show the prospective individual muscles of the ventrolateral body wall. Later at E5 (HH‐stage 27/28), Engert describes the external oblique muscle to be the first to split off the lateral side of the common abdominal muscle mass in the anterior lumbar region. During E6 and E7, Engert observed the separation of transversus muscle and internal oblique muscle within the abdominal muscle mass, and at E8, he states individual muscle formation as complete, including the rectus muscle which he holds to emerge from the ventralmost tip of the myotome as late as E8 (HH‐stage 34).

At variance with these earlier studies, we observed fiber reorientation in the transverse and external oblique anlagen earlier than previously reported, already at HH‐stage 26 (early E5), and we identified the rectus muscle earlier than noted previously, concomitant with the lateral muscles directly from the beginning of muscle splitting at HH‐stage 28 (late E5/E6). An explanation for this discrepancy might be that fiber orientation is better visible in whole mounts than in sections, and that for the same reason, the rectus muscle has initially been misidentified as part of the internal oblique muscle.

The same mode of development, with a ventrally expanding hypaxial myotome forming initially a uniform blastema, and subsequently splitting into individual muscle layers, is seen in rat embryos and appears to be a general concept in amniotes. In rats, muscle splitting starts at E14/E15 (Rizk & Adieb, 1982). The ingression of somatopleural mesenchyme between the individual muscles is a crucial step in muscle morphogenesis, as a number of experiments have shown that the splitting and arrangement of primitive muscle masses into distinct anatomical muscles is not an intrinsic property of the muscles but depends on signals from the neighboring mesenchyme and connective tissue fibroblasts (Chevallier & Kieny, 1982; Kardon et al., 2002, 2003; Stopak & Harris, 1982). Our experiments underline that the chicken abdominal wall is a useful model system to study muscle splitting in amniote embryogenesis.

### 4.3 Segmental origin of abdominal muscles

The segmental origin of the abdominal musculature has been addressed by a number of studies using different techniques of cell tracking. However, the results remained inconsistent, ranging from two segments (somites 26 and 27, Seno, 1961), or three segments (somites 27–29, Chevallier, 1979), to four segments of origin (somites 26–29, Rees et al., 2003) with variant segment numbers. In our study, we showed that abdominal muscles originate from at least five segments, somites 24, 25, 26, 27, and 28. The discrepancy between the earlier studies and the recent investigation could have several reasons. Different results concerning segment numbers could arise from different counting methods used which can easily produce systematic errors. In this study, we have confirmed our somite counting method using criteria based on the established system of segment identity by Burke et al. (1995), which is in accordance with Hox gene expression data. We therefore argue that our data are reliable and variant segment numbers found in earlier studies might need to be shifted accordingly. Furthermore, our use of muscle‐specific vectors in whole‐mount embryos, and our approach to analyze medial views of the abdominal wall, allows the identification of even small numbers of fibers which might have escaped attention in other studies.

Regarding the distribution of muscle fibers of different segmental origin in the abdominal muscle sheets, we found striking differences between specific muscles. In the transversus muscle, the segmental origin is in principle conserved, with more cranial segments contributing to the more cranial muscle portions, and more caudal segments contributing to more caudal muscle parts. Nevertheless, there is considerable overlap and no clear segmental borders in the muscle. By contrast, in the rectus muscle, the fibers of different origin appear randomly mixed. It is interesting to note that the transversus fibers have switched their orientation, rotating from longitudinal (craniocaudal) in the late myotomes to dorsoventral in the differentiated muscle. It appears that these reorienting fibers stay close to their original position, thus creating a semi‐segmental fiber pattern in the muscles. By contrast, the rectus fibers do not rotate but maintain the original longitudinal orientation of the myotomal fibers. It is likely that this is due to elongation of muscles fibers from different segments intercalating between each other until the length of the muscle is reached, thus creating a mosaic pattern of segmental origin in this muscle. In line with this hypothesis, Chevallier (1979) who investigated the origin of back muscles has found that while the short intrinsic back muscles maintain their segmental organization, the long, segment‐crossing intrinsic back muscles originate from all somites of the segments covered. The mechanism of segment‐crossing and axial elongation of muscle fibers is still unknown.

It is an interesting fact that the segmental origin of abdominal muscles and hindlimb muscles is partially overlapping. According to Chevallier who found abdominal muscles arising from somites 27 to 29, leg muscles arise from somites 26 to 32 (Chevallier, 1979), and according to Rees et al. (2003) who found abdominal muscles arising from somites 26 to 29, leg muscles arise from somites 26 to 33. In our data, even though we did not analyze leg muscle fate, we also routinely observed co‐staining of abdominal muscles and leg muscles after single somite labelling in segments 27 and 28 (see e.g., Figures 4 and 5). This means that these somites which contribute to abdominal muscles also give rise to leg muscles. This observation causes a conceptual problem because classically the abdominal muscles are considered as derivatives of ventrally and craniocaudally extending hypaxial myotomes. By contrast, limb muscles are known to be formed from individually emigrating muscle precursor cells which detach from the dissolving ventrolateral lips of the dermomyotomes and migrate into the limb buds prior to muscle fiber formation (reviewed in Scaal & Marcelle, 2018). It therefore seems that the hypaxial compartment at hindlimb level has a dual muscular fate, on the one hand, hypaxial myotomes forming the abdominal wall muscles, and on the other hand, hypaxial migratory muscle progenitors forming the leg muscle masses (Chevallier, 1979; Rees et al., 2003; Seno, 1961). Surprisingly, experimentally induced ectopic limbs at thoracic level recruit most hypaxial cells to the limbs, whereas distal ribs, distal intercostal muscles, and abdominal muscles do not properly form at the levels of surgery, so that this dual mode of muscle formation does not seem to apply to thoracic levels (Liem & Aoyama, 2009). At lumbosacral levels, this awaits further investigation. Presently nothing is known about the cellular and molecular mechanisms enabling this dual muscle program in the hypaxial somites.

Another cue to the origin of muscles is their innervation. It is generally assumed that the segmental origin of nerve fibers along the spinal cord is linked to the segmental origin of the respective muscles innervated. In human anatomy, the abdominal wall musculature is mainly innervated by intercostal nerves, that is, nerves originating in thoracic segments. In the textbook literature, there is disagreement about the exact segments contributing to abdominal muscle innervation, but there is consensus that several caudal thoracic segments as well as the subcostal nerve are involved. In addition, except for the external oblique and the rectus abdominis, the human abdominal muscles receive innervation from the iliohypogastric as well as the ilioinguinal nerves, which in most cases originate in Th12 and/or L1 (Klaassen et al., 2011; Standring, 2021). In birds, a similar pattern of innervation, which implies several intercostal nerves as well as the ilioinguinal and the iliohypogastric nerve from L1, has been reported in classical literature (Gadow, 1891) and adopted to textbooks (König & Liebich, 2001; Schummer, 1973), but more recent studies and exact data concerning the thoracic segments involved are lacking. Cell lineage studies using the quail‐chick chimerization technique have revealed that, in the avian hindlimb, the spinal segmental origin of motoneurons is in most cases corresponding to the segment number of somites which participate in the formation of a specific anatomical muscle (Lance‐Jones, 1988). For the abdominal muscles, our data predict that innervation includes at least the two caudalmost intercostal nerves (segments 24 and 25), the subcostal nerve (segment 26), as well as contributions of the ilioinguinalis and iliohypogastric nerves (segments 27 and 28), thus being in line with textbook data, but experimental confirmation of this hypothesis is still pending.

### 4.4 Muscle fiber elongation

The transition of segmental, mononucleate myotomal fibers into long, multinucleated definitive muscle fibers, as well as the reorientation of muscle fibers during axial muscle formation, is not well understood. Experiments by Gros et al. (2005) in the chicken embryo and Deries et al. (2010) in the mouse indicate that during epaxial muscle formation, myotomal fibers receive myonuclei from Pax7‐positive precursor cells and elongate into segment‐crossing muscle fibers. In the chick, these Pax7‐positive cells originate in the central dermomyotome (Gros et al., 2005). Splitting of muscle masses into distinct muscles, as well as reorientation of fibers from initially strict craniocaudal arrangement in the myotome to the oblique fibers of, for example, transversospinal muscles, depends on the influence of neighboring Scx‐positive connective tissue cells (Deries et al., 2010).

The mechanism of muscle cell fusion during the formation of muscle fibers in the chicken embryo has been investigated by Marcelle and coworkers (Sieiro‐Mosti et al., 2014). They found that epaxial trunk muscles are formed to the largest part by resident muscle precursor cells immigrating into the myotome from the central dermomyotome, that is, by secondary myotomal cells, which fuse with each other to form multinucleate fibers. The primary myotomal cells, which arise from the dermomyotomal lips and are the first to form the myotomal compartment, also form multinucleate fibers at later stages, even though with a less important contribution to the emerging muscles. They also become multinucleate by fusion with each other, and not, or rarely, with resident muscle precursor cells.

The study by Sieira‐Mosti et al. was limited to the segmental myotomes and did not address the formation of long muscle fibers which extend beyond the original segment borders. Our results in the abdominal musculature suggest that long muscle fibers only arise from fusion of cells within a given segment, and that no precursors of neighboring segments fuse to elongating fibers even when these intermingle during segment‐crossing growth. How this conservative mode of growth is mechanistically implemented is unknown.

### 4.5 Perspectives

Our work proposes the abdominal musculature in the chicken embryo as a suitable model system to study the formation of large, non‐segmental muscles from segmental anlagen in the hypaxial domain, as well as muscle splitting and muscle fiber orientation. The molecular and cellular mechanisms of these processes are still largely unknown, both in hypaxial abdominal and epaxial back muscles. As a starting point for further studies, we provide a systematic description of chicken abdominal muscle morphology. Our work thus provides a basis for further studies regarding the postulated dual fate of hypaxial muscle precursor cells towards abdominal wall and limb muscle fates, about the cellular and molecular mechanisms of segment‐crossing of myotome‐derived muscle fibers, and the process of muscle fusion in long fiber systems.

## AUTHOR CONTRIBUTIONS

Margarethe Draga: contributions to concept/design, acquisition of data, data analysis/interpretation, drafting of the manuscript, critical revision of the manuscript, and approval of the article. Luke Orth: acquisition of data, data analysis/interpretation, critical revision of the manuscript, and approval of the article. Julia Khabyuk: contributions to concept/design, acquisition of data, data analysis/interpretation, critical revision of the manuscript, and approval of the article. Vanessa Holtwick: acquisition of data, data analysis/interpretation, critical revision of the manuscript, and approval of the article. Johanna Heinen: acquisition of data, data analysis/interpretation, critical revision of the manuscript, and approval of the article. Felicitas Pröls: contributions to concept/design, data analysis/interpretation, critical revision of the manuscript, and approval of the article. Martin Scaal: contributions to concept/design, data analysis/interpretation, drafting of the manuscript, critical revision of the manuscript, and approval of the article.

## Data Availability

The data that support the findings of this study are available on request from the corresponding author. The data are not publicly available due to privacy or ethical restrictions.
